# The skeletal phenotype of achondrogenesis type 1A is caused exclusively by cartilage defects

**DOI:** 10.1242/dev.156588

**Published:** 2018-01-01

**Authors:** Ian M. Bird, Susie H. Kim, Devin K. Schweppe, Joana Caetano-Lopes, Alexander G. Robling, Julia F. Charles, Steven P. Gygi, Matthew L. Warman, Patrick J. Smits

**Affiliations:** 1 Orthopaedic Research Laboratories, Department of Orthopaedic Surgery, Boston Children's Hospital, Boston, MA 02115, USA; 2 Department of Cell Biology, Harvard Medical School, Boston, MA 02115, USA; 3 Department of Anatomy and Cell Biology, Indiana University, Indianapolis, IN 46202, USA; 4 Department of Medicine, Brigham and Women's Hospital, Boston, MA 02115, USA; 5 Howard Hughes Medical Institute, Boston Children's Hospital, Boston, MA 02115, USA; 6 Department of Genetics, Harvard Medical School, Boston, MA 02115, USA

**Keywords:** GMAP-210, Achondrogenesis type 1A, Conditional knockout, Golgin, Cartilage, Proteomics

## Abstract

Inactivating mutations in the ubiquitously expressed membrane trafficking component GMAP-210 (encoded by *Trip11*) cause achondrogenesis type 1A (ACG1A). ACG1A is surprisingly tissue specific, mainly affecting cartilage development. Bone development is also abnormal, but as chondrogenesis and osteogenesis are closely coupled, this could be a secondary consequence of the cartilage defect. A possible explanation for the tissue specificity of ACG1A is that cartilage and bone are highly secretory tissues with a high use of the membrane trafficking machinery. The perinatal lethality of ACG1A prevents investigating this hypothesis. We therefore generated mice with conditional *Trip11* knockout alleles and inactivated *Trip11* in chondrocytes, osteoblasts, osteoclasts and pancreas acinar cells, all highly secretory cell types. We discovered that the ACG1A skeletal phenotype is solely due to absence of GMAP-210 in chondrocytes. Mice lacking GMAP-210 in osteoblasts, osteoclasts and acinar cells were normal. When we inactivated *Trip11* in primary chondrocyte cultures, GMAP-210 deficiency affected trafficking of a subset of chondrocyte-expressed proteins rather than globally impairing membrane trafficking. Thus, GMAP-210 is essential for trafficking specific cargoes in chondrocytes but is dispensable in other highly secretory cells.

## Abstract (precis)

**Summary:** Conditional inactivation of the cis-Golgin GMAP-210 reveals that the skeletal phenotype in achondrogenesis type-1A, which is caused by mutations in GMAP-210, is solely due to impaired protein trafficking by chondrocytes.

## INTRODUCTION

The evolution of membrane-bound intracellular compartments in eukaryotic cells enabled the efficient separation of cell functions, e.g. nucleus for DNA replication, mitochondria for respiration, and endoplasmic reticulum (ER) for protein synthesis and folding. It also created the need for communication between different subcellular compartments. This interaction, and the interaction between the cell and its external environment, is mainly achieved through membrane trafficking. Membrane trafficking is a basic, life-essential function performed by all eukaryotic cells. It is essential for maintaining cellular homeostasis and plays a role in most, if not all, biological processes. A typical membrane trafficking event contains four steps (Bonifacino and Glick, 2004; Cai et al., 2007; Pfeffer, 2007): (1) cargo selection and the formation of transport vesicles at the donor compartment (Bonifacino and Lippincott-Schwartz, 2003; D'Arcangelo et al., 2013; Faini et al., 2013; Jackson, 2014; Kirchhausen, 2000; Kirchhausen et al., 2014; McMahon and Mills, 2004; Venditti et al., 2014); (2) vesicle delivery to the target compartment by diffusion or by motor-mediated transport along a cytoskeletal track (Fokin et al., 2014; Hancock, 2014; Hendricks et al., 2010; Roberts et al., 2013; Tumbarello et al., 2013); (3) tethering/capturing of the vesicle at the target compartment (Balderhaar and Ungermann, 2013; Brown and Pfeffer, 2010; Chia and Gleeson, 2014; Munro, 2011); and (4) fusion of the vesicle membrane with the target membrane, releasing the cargo into the lumen of the target compartment (Hong and Lev, 2014; Jena, 2011).

Insights into membrane trafficking have come from work performed in cell lines and in single-cell organisms such as yeast. However, these systems do not encompass the diversity of cell types present in multicellular organisms and may not model what occurs *in vivo* (Apodaca and Brown, 2014). These limitations are relevant to the autosomal recessive skeletal dysplasia achondrogenesis type 1A (ACG1A), which we previously reported is caused by loss-of-function mutations in *TRIP11* (Smits et al., 2010). Although *TRIP11* (thyroid receptor interacting protein 11) was initially considered a potential co-activator of thyroid hormone nuclear receptor β (Lee et al., 1995), subsequent studies provided strong evidence for its role in membrane trafficking. The protein was re-named GMAP-210 (Golgi-microtubule-associated protein of 210 kDa), as it binds microtubuli and localizes to the Golgi apparatus (GA) (Infante et al., 1999; Ramos-Morales et al., 2001). The GA, which is made of stacks of flattened membranous cisternae, is the main protein-sorting and post-translational modification center of a eukaryotic cell. It receives cargo (mainly from the ER) on its cis-side, proteins then acquire their post-translational modifications while moving through the medial Golgi, before being sorted into transport vesicles, destined for specific destinations in or outside the cell, at the trans-Golgi. As GMAP-210 contains a central coiled-coil domain and a GRAB (GRIP related ARF1 binding) domain, it has been classified as a member of the Golgin protein family (Infante et al., 1999; Ramos-Morales et al., 2001). Golgins function as tethering factors, capturing transport vesicles and aiding their fusion with their target organelles (Gillingham and Munro, 2016; Witkos and Lowe, 2017). Knockdown experiments using small interfering RNAs implicate GMAP-210 in ER-to-Golgi transport (Roboti et al., 2015). This role in ER vesicle tethering was elegantly demonstrated by directionally localizing GMAP-210 from the Golgi to the mitochondria. This resulted in the redirection of ER-derived vesicles to this organelle (Wong and Munro, 2014). Golgins also function to maintain the organization of the Golgi, and *in vitro* studies have shown that GMAP-210 plays an essential role in maintaining Golgi structure (Rios et al., 2004).

The lack of early embryonic lethality and the predominantly skeletal phenotype in humans and mice missing GMAP-210 was surprising as the protein is ubiquitously expressed and thought to be essential for cell function based on *in vivo* studies (Follit et al., 2008; Smits et al., 2010). We found that many cell types in GMAP-210-deficient mice had normal-appearing Golgi. However, we did observe massive ER swelling and precocious cell death in growth-plate chondrocytes along with impaired bone formation. Because bone formation (i.e. ossification) depends on cartilage formation, we could not determine whether the bone defect was cell autonomous or secondary to the cartilage defect. In addition, the perinatal lethality that occurs in GMAP-210-deficient humans and mice precludes the assessment of the postnatal roles of the protein in other tissues. Thus, it could not be determined, in global deficiency humans and mice, whether GMAP-210 is essential in cells that produce abundant extracellular matrix or that secrete large volumes of cargo, and whether chondrocytes use GMAP-210 to traffic all extracellular matrix proteins or only a subset of cargoes.

To address these aforementioned issues, we generated mice carrying conditional *Trip11*-null alleles. Herein, we detail studies in which we inactivated *Trip11 in vivo* specifically in chondrocytes, osteoblasts, the osteoclast encompassing hematopoietic lineage and exocrine pancreatic acinar cells. We also inactivated *Trip11* in primary cultured chondrocytes *in vitro* and used proteomics to determine whether chondrocytes require GMAP-210 for all extracellular matrix proteins or only a subset of cargoes. We found that *Trip11* inactivation in chondrocytes replicates the ACG1A phenotype, whereas there is no apparent phenotype when GMAP-210 is absent in osteoblasts, osteoclasts or pancreatic acinar cells. Our data demonstrate that the skeletal phenotype of ACG1A is caused exclusively by chondrocyte defects and that *Trip11* is dispensable in several other cell types that extensively use the membrane-trafficking machinery. Furthermore, we found that absence of GMAP-210 does not lead to intracellular accumulation of all secreted proteins, but only affects the secretion of a select group of cartilage extracellular matrix proteins.

## RESULTS

### The *Trip11*^cko^ allele is a true loss-of-function allele after Cre recombination

**Fig. 1.**
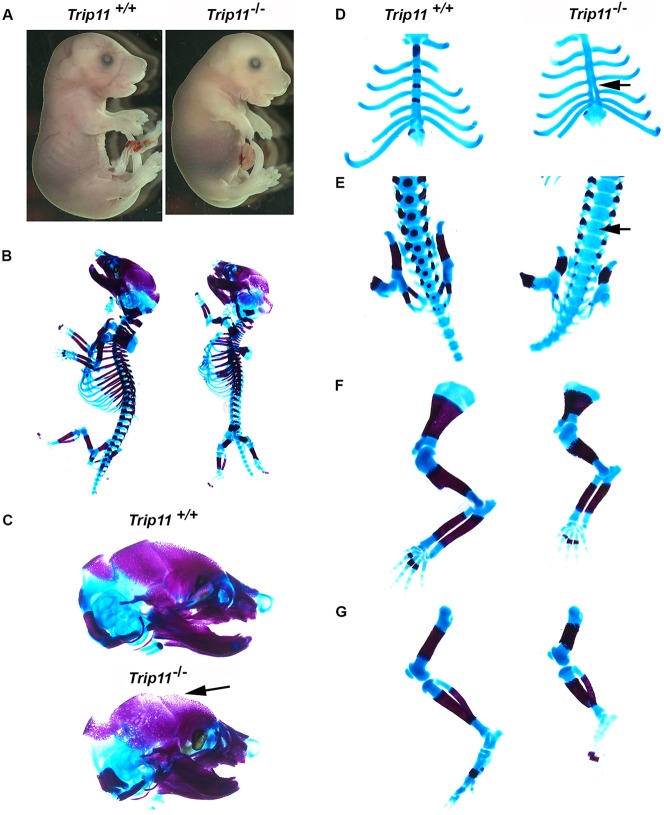
**Mice homozygous for the recombined *Trip11* conditional allele (*Trip11^−^*) have a severe skeletal dysplasia.** (A) E17.5 wild-type (*Trip11^+/+^*) and recombined (*Trip11^−/−^*) embryos. Note the short limbs, short snout, domed skull, protruding tongue and omphalocele in the recombined (i.e. knockout) embryo. (B-G) Skeletal preparations of the embryos shown in A. Compared with the *Trip11^+/+^* embryo, the *Trip11^−/−^* embryo has: (B) a smaller ribcage; (C) decreased mineralization of the calvarium (arrow); (D) absent mineralization of the sternum (arrow); (E) absent mineralization of the vertebral bodies (arrow); (F) short forelimbs; and (G) short hindlimbs. *n*=3; one representative result is shown.

Animals homozygous for the conditional allele (i.e. *Trip11*^cko/ck*o*^) were indistinguishable from wild-type and heterozygous (*Trip11*^cko/+^) mice, indicating that the inserted LoxP sites did not impair *Trip11* expression. In contrast, when the conditional allele was Cre recombined (i.e. *Trip11*^−^) and transmitted via the germline, homozygous *Trip11*^−/−^ offspring had the same skeletal dysplasia previously observed in mice with other loss-of-function alleles (Fig. 1A), featuring a short trunk, short limbs and a domed skull with a protruding tongue. Skeletal preparations of E17.5 *Trip11*^−/−^ embryos confirmed the smaller ribcage, shorter vertebral column and reduced length of the appendicular bones. Similar to ACG1A, a delay in mineralization of the vertebral column, sternum and the intramembranous skull bones was present in *Trip11*^−/−^ embryos (Fig. 1B-G).

**Fig. 2.**
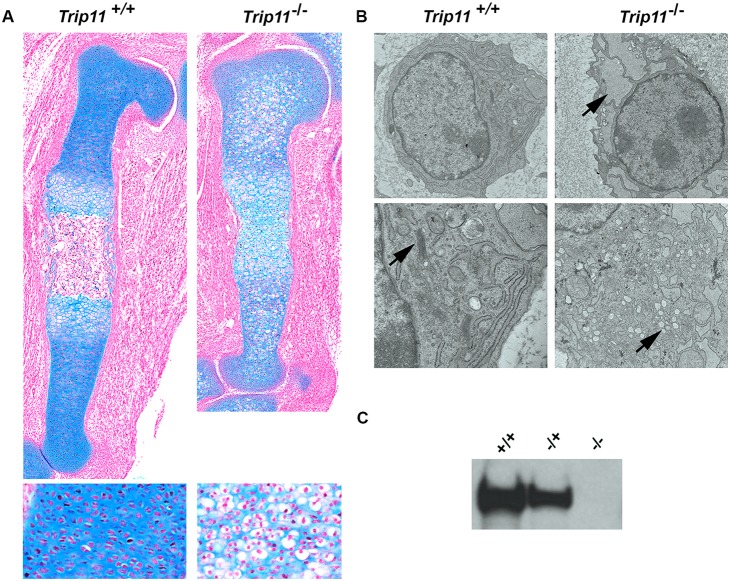
**Swelling of ER cisternae and disruption of the Golgi stack structure in chondrocytes of mice homozygous for a recombined *Trip11* conditional allele (*Trip11^−^*).** (A) Alcian Blue-stained sections through the humeri of E15.5 wild-type (*Trip11^+/+^*) and knockout (*Trip11^−/−^*) embryos. Note the delay in the formation of the primary ossification center in the knockout. Bottom panels: higher magnification of columnar chondrocytes. Note the swollen appearance of chondrocytes in the knockout. (B) Transmission electron microscopy pictures of epiphyseal chondrocytes from the humeri of E15.5 wild-type and knockout embryos (original magnifications: top, 2900×; bottom, 9300×). Note the increased size of ER cisternae in the knockout chondrocytes (top panel, arrow) and the disruption of the Golgi stack structure (bottom panels, arrow). (C) Western blot with a GMAP-210-specific antibody on the cell lysates of mouse embryonic fibroblasts extracted from E13.5 wild-type, *Trip11^−/+^* and knockout embryos. Note the complete absence of GMAP-210 protein in the knockout (−/−) cell lysate. *n*=3; one representative result is shown.

Alcian Blue-stained sections through the humeri of E15.5 mutant embryos showed a delay in the formation of the primary ossification center (Fig. 2A). Furthermore, chondrocytes in mutant embryos had a swollen appearance, with massive expansion of the ER and disruption of the Golgi apparatus (GA) evident by electron microscopy (EM) (Fig. 2B). Cell lysates obtained from mouse embryonic fibroblasts generated from E13.5 *Trip11^−/−^* embryos lacked immunodetectable GMAP-210, compared with *Trip11^+/+^* and *Trip11^+/−^* littermates (Fig. 2C). These data indicate that the *Trip11*^cko^ allele becomes a loss-of-function allele after Cre-recombination.

### Specific inactivation of *Trip11* in cartilage using *Col2a1*-Cre yields a phenotype identical to achondrogenesis type 1A

**Fig. 3.**
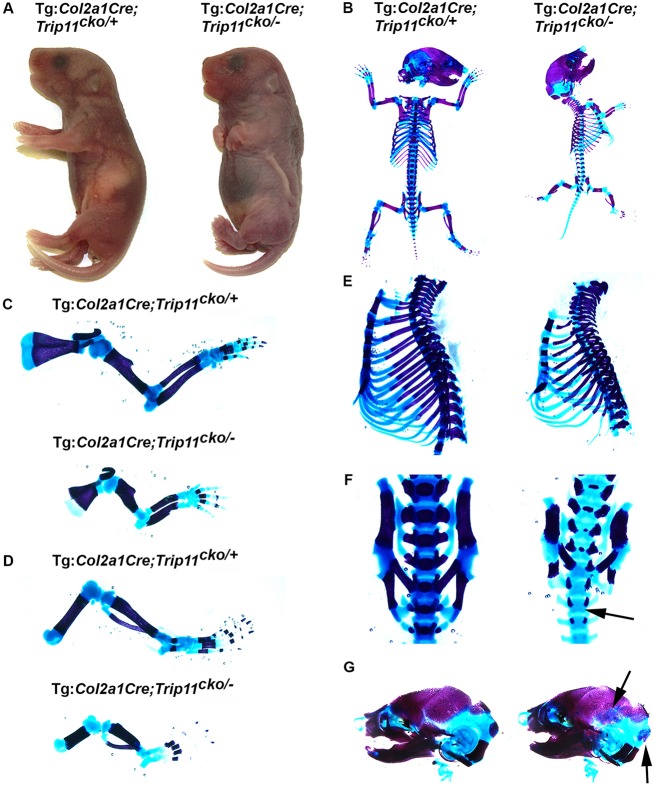
**Inactivation of *Trip11* in chondrocytes recapitulates the skeletal dysplasia seen in germline knockout mice.** (A) Control (Tg:*Col2a1*-Cre;*Trip11*^cko/+^) and chondrocyte knockout (Tg:*Col2a1*-Cre;*Trip11*^cko/−^) newborn pups. Note the short snout, domed skull and short limbs in the chondrocyte knockout. (B-G) Skeletal preparations of the pups shown in A. Compared with the control embryo, the embryo with the chondrocyte-specific deletion of *Trip11* has: short (C) forelimbs and (D) hindlimbs; (E) a small ribcage; (F) delayed mineralization of the vertebral body (arrow); and (G) decreased mineralization of the skull (arrows). *n*=3; one representative result is shown.

Mice lacking GMAP-210 in chondrocytes were generated using the *Col2a1*-Cre transgene. Tg:*Col2a1*-Cre;*Trip11*^cko/−^;*ROSA26*^mTmG/+^ newborn mice were present at the expected Mendelian frequency and died shortly after birth with a severe chondrodysplasia similar to that observed in *Trip11^−/^*^−^ mice (Fig. 3A). Newborn Tg:*Col2a1*-Cre;*Trip11*^cko/−^;*ROSA26*^mTmG/+^ pups had short limbs, a short snout and a domed skull. Skeletal preparations revealed shorter bones in the extremities, a smaller ribcage, and a delay in mineralization of the vertebral column and the skull bones (Fig. 3B-G).

**Fig. 4.**
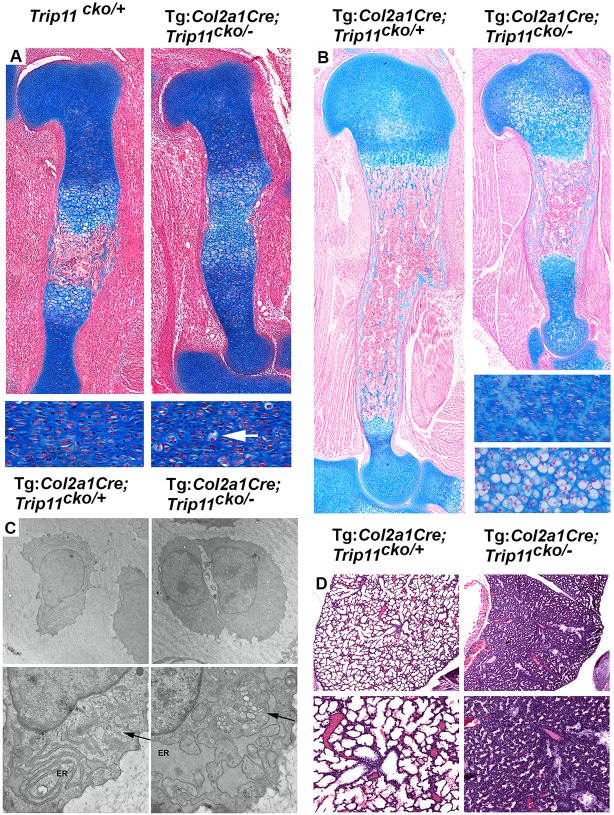
**Specific inactivation of *Trip11* in chondrocytes causes swelling of ER cisternae and disruption of the Golgi stack structure.** (A) Alcian Blue-stained sections through the humeri of E15.5 control (*Trip11^cko/+^*) and chondrocyte knockout (Tg:*Col2a1*-Cre;*Trip11*^cko/−^) embryos. Bottom panels show higher magnification of columnar chondrocytes, indicating the delay in the formation of the primary ossification center and the swollen appearance of some chondrocytes in the humerus of the chondrocyte knockout (arrow). (B) Alcian Blue staining of sections through the humerus of P0 control (Tg:*Col2a1*-Cre;*Trip11*^cko/+^) and chondrocyte knockout (Tg:*Col2a1*-Cre;*Trip11*^cko/−^) pups. Lower right panels show higher magnification of columnar chondrocytes. Note the swollen appearance of chondrocytes in the humerus of the chondrocyte knockout. (C) Transmission electron microscopy pictures of epiphyseal chondrocytes from the humerus of P0 control (Tg:*Col2a1*-Cre;*Trip11*^cko/+^) and chondrocyte knockout (Tg:*Col2a1*-Cre;*Trip11*^cko/−^) pups (original magnifications: top, 2900×; bottom, 9300×). Note the increased size of ER cisternae in the chondrocyte knockout (ER) and the disruption of the Golgi stack structure (arrow). (D) Hematoxylin staining of sections through the lungs of P0 control (Tg:*Col2a1*-Cre;*Trip11*^cko/+^) and chondrocyte knockout (Tg:*Col2a1*-Cre;*Trip11*^cko/−^) pups. Higher magnification of the alveoli is shown in the bottom panels. Note the impaired alveolar development in the mutant lung. *n*=3; one representative result is shown.

Histology analysis of the humeri of E15.5 mutant embryos demonstrated delayed formation of the primary ossification center and areas where columnar chondrocytes were swollen (Fig. 4A). At P0, widespread chondrocyte swelling was observed in the humeri of mutant pups; furthermore, hypertrophic zones were absent and the humeri featured an abnormally thick bone collar (Fig. 4B). Electron microscopy revealed that P0 mutant humeral chondrocytes had ER swelling and loss of GA stacking (Fig. 4C). Interestingly, decreased lung alveolar formation, which had been observed in mice with global loss-of-function mutations in *Trip11* (Follit et al., 2008; Smits et al., 2010), was also observed in Tg:*Col2a1*-Cre;*Trip11*^cko/−^;*ROSA26*^mTmG/+^ mice (Fig. 4D). Thus, the alveolar phenotype appears secondary to the small ribcage, rather than a primary lung cell phenotype. The American Thoracic Society has reported that alveolar deficiency has been observed in other conditions characterized by insufficient thoracic volume (American Thoracic Society, 2004). The alveolar insufficiency is the likely cause of death of Tg:*Col2a1*-Cre;*Trip11*^cko/−^;*ROSA26*^mTmG/+^ mice. When combined, these data show that the skeletal phenotype of cartilage-specific *Trip11*-null mice is identical to that of global *Trip11*-null mice.

### Absence of GMAP-210 does not impair osteoblast and osteoclast function

Humans and mice with global deficiency of GMAP-210 have significantly reduced ossification of vertebral bodies and skull bones. That both endochondral (vertebral bodies) and intramembranous bones (skull) show defects, implies a direct role for GMAP-210 in bone formation. However, a delay in mineralization of these skeletal elements is also observed in cartilage-specific *Trip11* knockout mice, suggesting that the delay is a secondary consequence of the cartilage phenotype. To investigate whether GMAP-210 has cell-autonomous functions in bone, we specifically inactivated *Trip11* in osteoblasts using the *Bglap-*Cre transgene and, as osteoclasts derive from the hematopoietic lineage, in hematopoietic stem cells using the *Vav1-*Cre transgene.

**Fig. 5.**
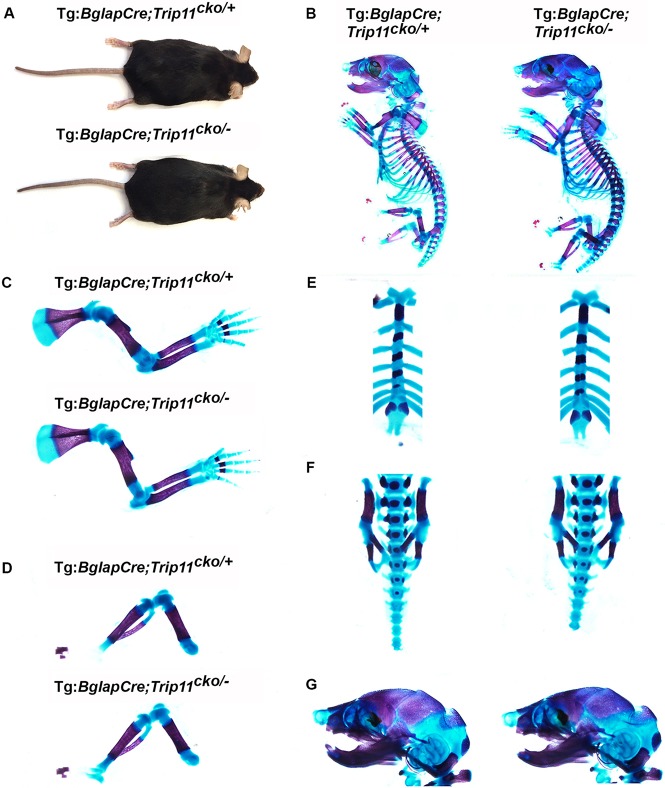
**Specific inactivation of *Trip11* in osteoblasts does not result in a skeletal dysplasia.** (A) Five-month-old control (Tg:*Bglap*-Cre;*Trip11*^cko/+^*;ROSA26*^mTmG*/+*^) and osteoblast knockout (Tg:*Bglap*-Cre;*Trip11*^cko/−^*;ROSA26*^mTmG*/+*^) mice are indistinguishable. (B-G) Skeletal preparations of E17.5 control (Tg:*Bglap*-Cre;*Trip11*^cko/+^*;ROSA26*^mTmG*/+*^) and osteoblast knockout (Tg:*Bglap*-Cre;*Trip11*^cko/−^*;ROSA26*^mTmG*/+*^) embryos. (B) Whole skeletal preparations show no size difference between control and osteoblast knockouts. (C-G) Higher magnifications of the forelimbs (C), hindlimbs (D), sternums (E), lumbar vertebrae (F) and skulls (G) showing no differences between control and osteoblast knockout mice. *n*=3; one representative result is shown.

**Fig. 6.**
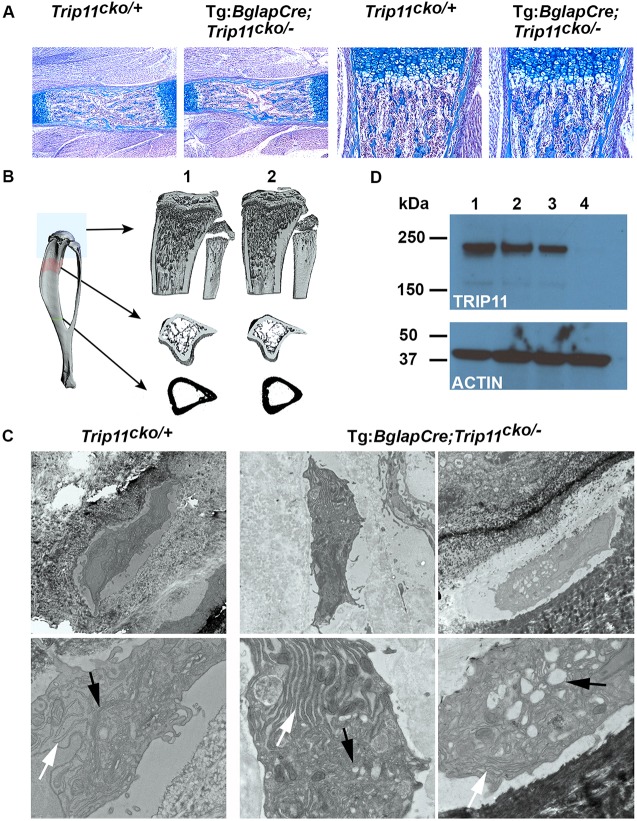
**Specific inactivation of *Trip11* in osteoblasts does not result in swelling of ER cisternae in their osteocyte descendants, but does disrupt Golgi stack structure.** (A) Alcian Blue-stained sections through the humeri of P0 control (*Trip11*^cko/+^*;ROSA26*^mTmG*/+*^) and osteoblast knockout (Tg:*Bglap*-Cre;*Trip11*^cko/−^*;ROSA26*^mTmG*/+*^) littermates. Left: low magnification of the primary ossification centers. Right: higher magnification of the primary spongosia of the proximal growth plates. No differences between control and osteoblast knockout are observed. (B) µCT of the tibias of 6-week-old control (1: *Trip11*^cko/+^*;ROSA26*^mTmG*/+*^) and osteoblast knockout (2: Tg:*Bglap*-Cre;*Trip11*^cko/−^*;ROSA26*^mTmG*/+*^) mice shows the mice have similar trabecular and cortical bone architecture. (C) Transmission electron microscopy pictures of calvarial osteocytes from P5 control (*Trip11*^cko/+^*;ROSA26*^mTmG*/+*^) and osteoblast knockout (Tg:*Bglap*-Cre;*Trip11*^cko/−^*;ROSA26*^mTmG*/+*^) pups. Original magnifications: 2900× (top); 9300× (bottom). Note the absence of swelling of ER cisternae in control and mutant osteocytes (white arrows), but that Golgi cisternae (black arrows) are increased in size and not organized into stacks in the mutant. (D) Western blot analysis of lysates from GFP-sorted primary calvarial outgrowth cultures established from 5-day-old control (Tg:*Bglap*-Cre;*Trip11*^cko/+^*;ROSA26*^mTmG*/+*^) and osteoblast knockout (Tg:*Bglap*-Cre;*Trip11*^cko/−^*;ROSA26*^mTmG*/+*^) mice. (Lane 1) GFP-negative control cells; (lane 2) GFP-positive control cells; (lane 3) GFP-negative osteoblast knockout cells; and (lane 4) GFP-positive osteoblast knockout cells. Note the absence of GMAP-210 protein in GFP-positive osteoblast knockout cells. Re-probing of the western blot with an anti-actin antibody serves as a loading control. *n*=3; one representative result is shown.

Tg:*Bglap*-Cre;*Trip11*^cko/−^*;ROSA26*^mTmG*/+*^ offspring were born at the expected Mendelian frequency and appeared normal (Fig. 5A). Skeletal preparations of E17.5 mutant and control embryos showed that *Trip11* inactivation in osteoblasts had no effect on the size or the mineralization of either endochondral or intramembranous bones (Fig. 5B-G). Histological sections of humeri from P0 osteoblast-specific knockout pups revealed similar amounts of trabecular and cortical bone when compared with control littermates (Fig. 6A). EM studies revealed no swelling of ER cisternae in mutant osteoblasts; however, they did feature an abnormal GA stack structure (Fig. 6C). Despite the abnormal GA, when analyzed at 6 weeks of age osteoblast-specific knockout mice had the same µCT bone measurements as their littermate controls (Fig. 6B and Fig. S1A).

To confirm that the *Bglap*-Cre transgene recombined the *Trip11^cko^* allele, we recovered cells from calvariae of P5 pups. We used the ROSA26*^mTmG^* allele to flow sort primary osteoblasts, which now fluoresce green, from non-osteoblasts, which fluoresce red. When western blots were immunodetected using an anti-GMAP-210 antibody, no protein was detected in osteoblasts from Tg:*Bglap*-Cre;*Trip11*^cko/−^*;ROSA26*^mTmG*/+*^ mice (Fig. 6D).

**Fig. 7.**
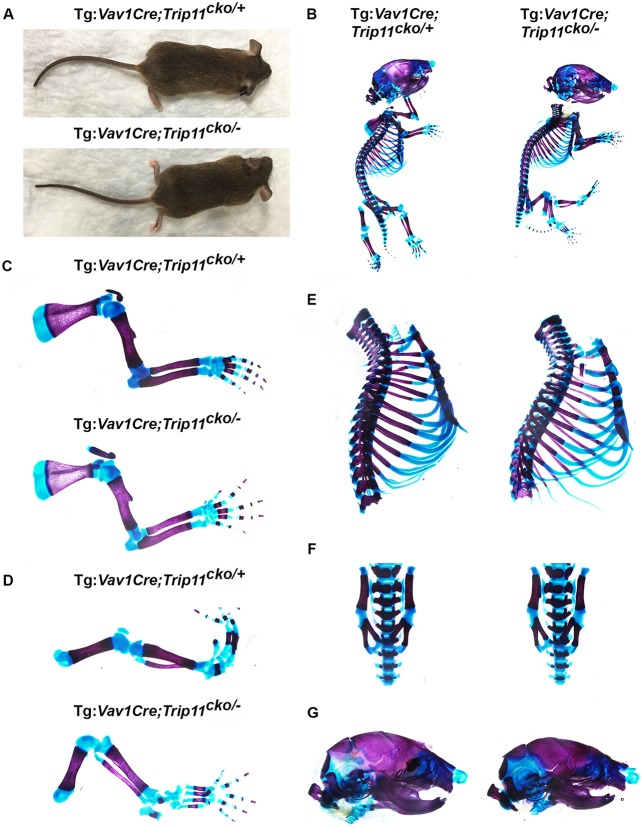
**Specific inactivation of *Trip11* in the hematopoietic lineage does not affect viability or cause skeletal dysplasia.** (A) Eight-week-old male control (Tg:*Vav1*-Cre;*Trip11*^cko/+^*;ROSA26*^mTmG*/+*^) and blood cell and osteoclast knockout (Tg:*Vav1*-Cre;*Trip11*^cko/−^*;ROSA26*^mTmG*/+*^) mice. No dwarfism is present in the blood cell and osteoclast knockout animal. (B-G) Skeletal preparations of P0 control (Tg:*Vav1*-Cre;*Trip11*^cko/+^*;ROSA26*^mTmG*/+*^) and blood cell and osteoclast knockout (Tg:*Vav1*-Cre;*Trip11*^cko/−^*;ROSA26*^mTmG*/+*^) newborns. (B) Whole skeletal preparations showing the mice are indistinguishable. (C-G) Higher magnifications of the: (C) forelimbs, (D) hindlimbs, (E) sternums, (F) lumbar vertebrae and (G) skulls showing no differences between control and blood cell and osteoclast knockout mice. *n*=3; one representative result is shown.

**Fig. 8.**
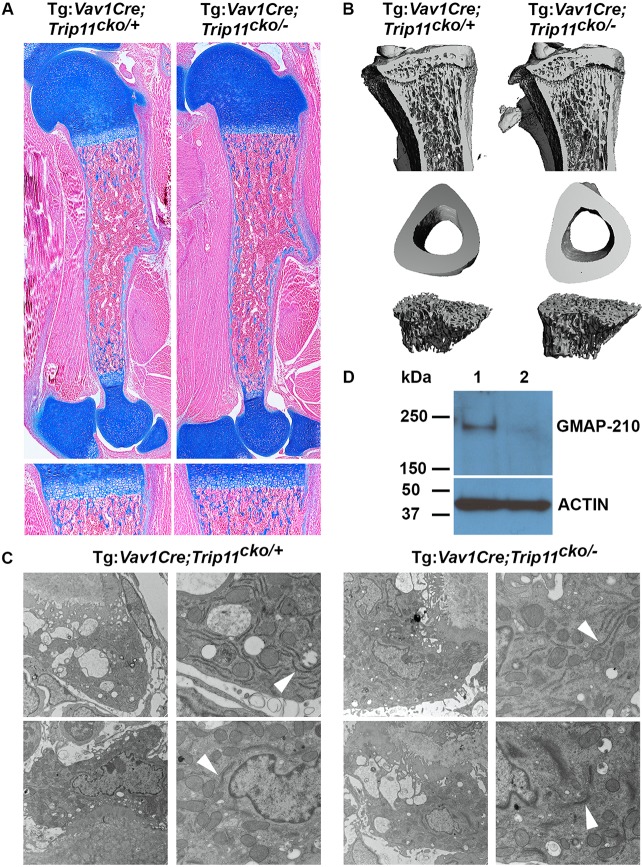
**Inactivation of *Trip11* in osteoclasts does not result in swelling of ER cisternae and does not disrupt Golgi stack structure.** (A) Alcian Blue-stained sections through the humeri of P0 control (Tg:*Vav1*-Cre;*Trip11*^cko/+^*;ROSA26*^mTmG*/+*^) and blood cell and osteoclast knockout (Tg:*Vav1*-Cre;*Trip11*^cko/−^*;ROSA26*^mTmG*/+*^) pups. Bottom: higher magnification of the primary spongosia of the proximal growth plates. No differences between mutant and control can be observed. (B) µCT of the tibias of 8-week-old control (Tg:*Vav1*-Cre;*Trip11*^cko/+^*;ROSA26*^mTmG*/+*^) and mutant (Tg:*Vav1*-Cre;*Trip11*^cko/−^*;ROSA26*^mTmG*/+*^) mice. No differences in the density of trabeculae or in the thickness of the bone collar can be observed between mutant and control mice. (C) Transmission electron microscopy pictures of tibial osteoclasts from 8-week-old control, and blood cell and osteoclast knockout mice. Original magnifications: 2900× (left); 9300× (right) Note the presence of normal ER cisternae (arrowheads, top panels) and normal Golgi (arrowheads, bottom panels) in both mice. (D) Western blot analysis of lysates of isolated bone marrow hematopoietic precursor cells from 8-week-old control (lane 1), and blood cell and osteoclast knockout (lane 2) mice. Note the absence of GMAP-210 protein in the knockout mice. Re-probing of the western blot with an anti-actin antibody serves as a loading control. *n*=3; one representative result is shown.

Mice lacking GMAP-210 in hematopoietic stem cell derivatives, which include osteoclasts (i.e. *Tg:Vav1-*Cre;*Trip11*^cko/−^*;ROSA26*^mTmG*/+*^), were born at the expected Mendelian frequency and appeared normal (Fig. 7A). Skeletal preparations of P0 mutant (*Tg:Vav1-*Cre;*Trip11*^cko/−^*;ROSA26*^mTmG*/+*^) and control pups (*Tg:Vav1-*Cre*;Trip11*^cko/+^*;ROSA26*^mTmG*/+*^) showed that *Trip11* inactivation throughout the hematopoietic lineage had no affect on the size or the mineralization of either endochondral or intramembranous bones (Fig. 7B-G). Furthermore, histological analysis of sections through the humeri of mutant and control P0 pups showed normal amounts of trabeculae, cortical bone and bone marrow (Fig. 8A). By EM, osteoclasts lacking GMAP-210 exhibited no swelling of the ER cisternae and had normal GA architecture (Fig. 8B). The absence of a bone phenotype was further confirmed by µCT on 8-week-old control and mutant mice (Fig. 8B and Fig. S1B).

Since the *Vav1-*Cre transgene is also active in immunoglobulin-producing lymphocytes, we analyzed IgG levels in serum from mutant and control 8-week-old mice. Coomassie Blue staining of SDS-PAGE separated serum did not reveal any differences in the IgG band intensity between mutant and control mice, implying that inactivation of *Trip11* does not impair IgG secretion (Fig. S2).

To confirm that the *Vav1-*Cre transgene recombined the *Trip11^cko^* allele, we isolated hematopoietic precursors from the bone marrow of mutant and control 8-week-old mice and extracted protein. A western blot using a GMAP-210 specific antibody showed the total absence of GMAP-210 in *Tg:Vav1-*Cre;*Trip11*^cko/−^*;ROSA26*^mTmG*/+*^ hematopoietic precursors (Fig. 8D). Together, these data show that absence of GMAP-210 does not interfere with either osteoblast or osteoclast function.

### A subset of secreted proteins is retained in GMAP-210-deficient chondrocytes

Massive expansion of ER cisternae is a prominent feature in GMAP-210-deficient chondrocytes. Chondrocytes produce large amounts of extracellular matrix proteins, so ER swelling could be a consequence of globally impaired membrane trafficking. However, osteoblasts, which also produce abundant extracellular matrices, have normal-appearing ER when GMAP-210 is absent. Similarly, the ER of the highly secretory osteoclasts is also unaffected by the absence of GMAP-210. Furthermore, when we inactivated *Trip11* specifically in the acinar cells of the adult pancreas, which secrete massive amounts of digestive enzymes, using the *Mist1-*CreERT2 transgene, we did not observe any swelling of the ER cisternae in these cells (Fig. S3).

**Fig. 9.**
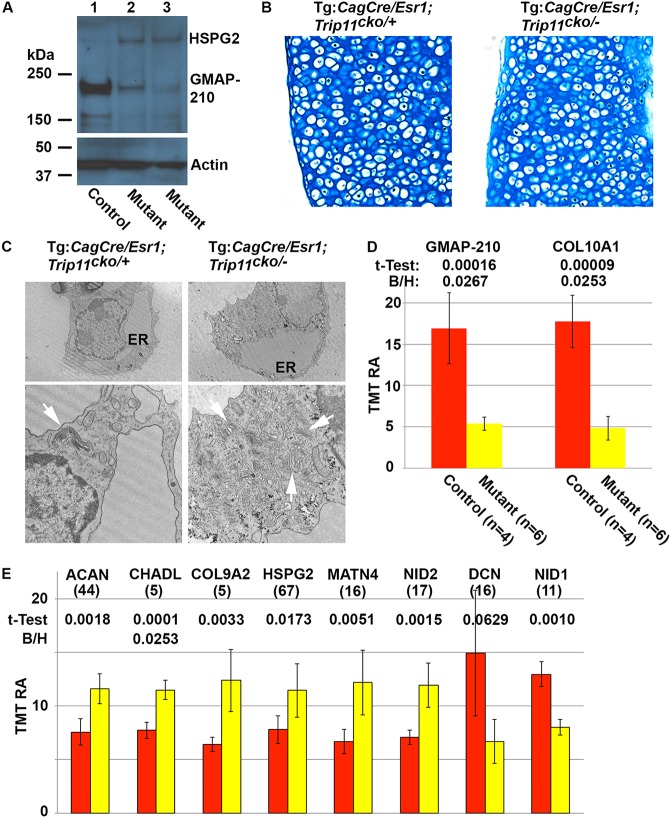
***Ex vivo* inactivation of *Trip11* in primary chondrocyte pellet cultures.** (A) Western blot of cell lysates from 4-OH tamoxifen-treated primary chondrocyte pellet cultures using antibodies against perlecan (HSPG2) and GMAP-210. Lysates come from control (lane 1, *Trip11^cko/cko^;ROSA26*^mTmG*/+*^) and from two different induced *Trip11* knockout (lanes 2 and 3) pellet cultures: (lane 2) Tg:*CagCre/Esr1;Trip11^cko/cko^;ROSA26*^mTmG*/+*^ and (lane 3) Tg:*CagCre/Esr1;Trip11^cko/−^;ROSA26*^mTmG*/+*^. Note the reduction in immunodetectable GMAP-210 and the increase in HSPG2 when *Trip11* was inactivated. Re-probing of the western blot with an anti-actin antibody serves as a loading control. (B) Alcian Blue-stained sections through 4-OH tamoxifen-treated control (Tg:*CagCre/Esr1;Trip11^cko/+^;ROSA26*^mTmG*/+*^) and *Trip11*-inactivated (Tg:*CagCre/Esr1;Trip11^cko/−^;ROSA26*^mTmG*/+*^) primary chondrocyte pellet cultures*.* Both control and *Trip11*-inactivated cultures produce abundant extracellular matrix. (C) Representative electron microscopy images of 4-OH tamoxifen-treated control and *Trip11*-inactivated primary chondrocyte pellet cultures*.* Original magnification: 4800× (top). Note both chondrocytes have enlarged endoplasmic reticulum (ER) cisternae. Original magnification: 9300× (bottom). Note the Golgi apparatus (white arrows) is enlarged and more disorganized in the *Trip11*-inactivated chondrocytes. (D) Abundance of GMAP-210 and COL10A1 in lysates from 4-OH tamoxifen-treated control (red) and *Trip11* inactivated (yellow) primary chondrocyte pellet cultures, as determined by tandem tag mass spectroscopy. Student's *t*-test adjusted *P*-value. Data are mean±s.d. TMT-RA, tandem mass tag relative abundance. (E) Graph depicting extracellular matrix proteins whose intracellular abundance increased or decreased in 4-OH tamoxifen-treated *Trip11*-inactivated (yellow; Tg:*CagCre/Esr1;Trip11^cko/−^;ROSA26*^mTmG*/+*^) versus control (red; (*Trip11^cko/cko^;ROSA26*^mTmG*/+*^) primary chondrocyte pellet cultures, as determined by tandem tag mass spectroscopy. CHADL, chondro-adherin like; HSPG2, perlecan; COL9A2, type 9 collagen α2 chain; ACAN, aggrecan; MATN4, matrillin 4; NID2, nidogen 2; NID1, nidogen 1; DCN, decorin. Data are mean±s.d. *n* values are in parentheses. Student's *t*-test adjusted *P* values and Benjamini-Hochberg FDR (B/H) (mutiple test) corrected *P* values are shown. GMAP-210, COL10A1 and CHADL are significantly different from control.

Therefore, to determine whether the ER retention in chondrocytes is due to impaired trafficking of specific proteins, we cultured primary chondrocytes from mice with conditional alleles, inactivated the alleles *ex vivo* and then compared these chondrocytes intracellular proteomes with those of controls. Pellet cultures of primary chondrocytes from Tg:*Cag*-Cre/Esr1;*Trip11*^cko/−^;*ROSA26*^mTmG/+^ mice and control littermates were treated with 4-OH tamoxifen for 2 weeks while they were actively producing cartilage matrices. We confirmed by western blot that GMAP-210 was depleted in the cells (Fig. 9A). Histological sections through the pellet cultures demonstrated that they contained differentiated chondrocytes that had produced a glycosaminoclycan-rich matrix that stains with Alcian Blue (Fig. 9B). Interestingly, by EM, experimental and control chondrocytes exhibited ER swelling. However, Golgi swelling and disappearance of the GA stack structure was evident only in the chondrocytes lacking GMAP-210 (Fig. 9C). For mice with global GMAP-210 deficiency, we previously observed that perlecan (HSPG2) was retained within their chondrocyte ER (Smits et al., 2010). Therefore, we performed western blotting to immunodetect perlecan in chondrocytes from the pellet culture experiments; we observed that perlecan levels also increased in chondrocytes in which *Trip11* expression had been inactivated *ex vivo* (Fig. 9A).

We next compared the proteomes of chondrocyte pellet cultures in which GMAP-210 had, or had not, been depleted *ex vivo*. A total of 7636 different proteins were detected (Table S1). Comparison between mutant and control cultures confirmed the reduction in GMAP-210 levels in mutant cultures and revealed a statistically significant (i.e. passing both Student's *t*-test and Benjamini-Hochberg FDR) reduction in COL10A1 protein levels, indicating that, similar to what we observed *in vivo*, chondrocytes lacking GMAP-210 fail to undergo hypertrophic differentiation *in vitro* (Fig. 9D). In addition to GMAP-210 and COL10A1, the intracellular levels of 62 proteins were significantly lower in GMAP-210-depleted chondrocytes, whereas the intracellular levels of 100 proteins were significantly higher in GMAP-210-depleted chondrocytes. Of the latter group, 39 proteins have an established or predicted role in membrane trafficking and/or Golgi/ER function (Table S2).

With regard to cartilage extracellular matrix proteins, only CHADL (chondroadherin-like protein) had a statistically significant increased intracellular protein abundance in GMAP-210-depleted chondrocytes after controlling for multiple hypothesis testing. However, using Student's *t*-test, levels of perlecan, COL9A2, aggrecan, matrillin 4 and nidogen 2 were found to be significantly increased in GMAP-210-depleted chondrocytes compared with controls, whereas nidogen 1 and decorin levels were significantly decreased (Fig. 9E). We confirmed the increased retention of perlecan and COL9A2 in GMAP-210-depleted cells using western blots (Fig. 9A and Fig. S4). Interestingly, the intracellular levels of many abundant cartilage extracellular matrix proteins, including COL2A1, COL9A1, fibromodulin, cartilage oligomeric matrix protein, biglycan, link protein, matrillin 3 and asporin, were not significantly affected by GMAP-210 depletion (Fig. S5A). Furthermore, the intracellular levels of more broadly expressed extracellular matrix proteins, including COL1A1 and laminins β1 and β2, were also unaffected. These findings show that inactivation of *Trip11*, despite impairing GA structure, does not result in a general secretion defect in chondrocytes, but instead affects the secretion of a specific set of proteins.

## DISCUSSION

Despite its importance for cellular function, most studies on membrane trafficking have been performed in yeast or cultured cells. Only recently have studies began to address this crucial process in complex environments such as tissues or multicellular organisms like fish and mammals. Thus, there is limited knowledge regarding cell type- and tissue-specific aspects of membrane trafficking. Tissue specificity in membrane trafficking is exemplified in the lethal skeletal dysplasia ACG1A, which is caused by mutations in *TRIP11*, the gene encoding the ubiquitously expressed cis-Golgin GMAP-210. Despite the ubiquitous expression of this vesicle-tethering factor, humans lacking GMAP-210 survive to birth with a phenotype that appears limited to the skeletal system. GMAP-210 has no closely related family members. Two other cis-Golgin proteins have been identified in the human and mouse genomes (GM-130 and Golgin-160), but both of these golgins are ubiquitously expressed, making compensation by other cis-Golgin proteins an unlikely explanation for the absence of a phenotype in other tissues (Munro, 2011). In this study, we have made use of mice carrying conditional *Trip11*-null alleles to gain insight into how tissue specificity is accomplished in ACG1A.

We found that inactivation of *Trip11* specifically in chondrocytes completely recapitulates the ACG1A phenotype. Tg:*Col2a1*-Cre;*Trip11*^cko/−^;*ROSA26*^mTmG/+^ mice display a severe and lethal skeletal dysplasia, the features of which are identical to ACG1A. This includes the delay in mineralization of the vertebral column and the bones of the skull, thus showing that these features are a secondary consequence of the cartilage phenotype and not a cell-autonomous bone phenotype. This was further confirmed by the finding that *Trip11* inactivation specifically in bone-forming osteoblasts or bone-resorbing osteoclasts (indirectly through inactivation in hematopoietic precursors) did not result in any detectable bone phenotype. Our data therefore show that the skeletal phenotype of ACG1A is caused exclusively by the absence of GMAP-210 in chondrocytes and that this ubiquitously expressed cis-Golgin is not essential in osteoblasts and osteoclasts.

Chondrocytes secrete a large volume of protein to form their abundant extracellular matrix. However, our data seem to exclude the possibility that GMAP-210 causes a general dysfunction in protein trafficking because other highly secretory cell types, such as osteoblasts, osteocytes, pancreatic acinar cells and B-lymphocytes, appear to function normally in the absence of GMAP-210. Thus, the effect of GMAP-210 deficiency more likely depends upon the type of cargo produced by a cell, rather than that cell's cargo volume. We addressed cargo specificity by comparing the proteomes of primary chondrocyte pellet cultures in which GMAP-210 was either absent or present. We found that lack of GMAP-210 results in the intracellular accumulation of proteins with an established or predicted role in membrane trafficking and/or Golgi/ER function, confirming that GMAP-210 depletion has a disruptive effect on the membrane-trafficking process. However, despite this, absence of GMAP-210 does not lead to a general secretion defect, but instead affects secretion of a subset of extracellular matrix proteins. The affected cargoes do not share a common feature; however, three of them (CHADL, aggrecan and COL9A2) are highly and specifically expressed by chondrocytes, with aggrecan being the most highly expressed protein in chondrocytes [using immunofluorescence microscopy, we previously did not observe intracellular retention of aggrecan *in vivo* (Smits et al., 2010); however, retention may have been masked by the extremely high abundance of aggrecan in cartilage]. Because of the chondrocyte-specific expression of these three proteins, it is tempting to conclude that their intracellular accumulation is mainly responsible for the ACG1A phenotype. Creating double-knockout (i.e. *Trip11/Chadl*, *Trip11/Acan* or *Trip11/Col9a2*) embryos and then looking for an improvement in the skeletal phenotype could be a way to conclusively assess whether intracellular accumulation of a specific cargo is responsible for the ACG1A phenotype.

Recently, multiple studies have investigated the mechanism by which GMAP-210 recognizes ER-derived vesicles. This would occur through its N-terminal amphipathic lipid packing sensor (ALPS) domain. The ALPS is a membrane-binding domain that is sensitive to membrane curvature, i.e. it is able to bind curved membranes, but has a much lower affinity for flat membranes. It allows GMAP-210 to distinguish between round small transport vesicles and larger flatter membrane structures such as Golgi cisternae (Drin et al., 2007, 2008; Wong et al., 2017). It has been suggested that the ALPS motif binds to the surface of lipid membranes by the random insertion of its hydrophobic residues into so-called lipid packing defects (i.e. voids) present in lipid membranes (Magdeleine et al., 2016; Vamparys et al., 2013; Vanni et al., 2013). The properties of the ER membrane (low sterol, low electrostatics and high level of unsaturation) in combination with a high curvature would lead to a high level of packing defects on ER-to-Golgi transport vesicles. This would enable efficient binding of these vesicles to GMAP-210 (Magdeleine et al., 2016; Vanni et al., 2013). As the membranes of vesicles derived from the trans side of the Golgi, or other organelles, have a different composition with fewer packing defects, they would not be recognized by GMAP-210 (Klemm et al., 2009; Magdeleine et al., 2016). Based on these studies, it was proposed that GMAP-210 sorts transport vesicles at the cis-Golgi according to size and lipid composition, but would not be able to distinguish vesicles containing different cargoes (Magdeleine et al., 2016). One would expect that if GMAP-210 functions as a broad filter for transport vesicles, its absence would lead to a general secretion defect. This is contradicted by our proteome data, which shows that only a select few cartilage extracellular matrix proteins accumulate intracellularly in the absence of GMAP-210. This suggests that mechanisms must exist through which GMAP-210 is capable of recognizing transport vesicles containing a specific cargo.

In summary, we have shown that the skeletal phenotype of ACG1A is caused exclusively by a chondrocyte defect. Furthermore, our data indicate that the defective cartilage is not the result per se of the high volume of cargo secreted by chondrocytes, but is instead a consequence of the role of GMAP-210 in the intracellular transport of a subset of cargo, which includes proteins specifically expressed by chondrocytes.

## MATERIALS AND METHODS

### Generation of mice carrying *Trip11* conditional knockout alleles

All animal experiments were carried out under protocols reviewed and approved by Children's Hospital Boston Institutional Animal Care and Use Committee, USA. A targeting vector, using pBluescript II KS(+) (Promega) as backbone, was constructed in which the first exon of *Trip11* (which contains the ATG) was flanked with LoxP sites (Fig. S5B). To avoid disrupting important upstream regulatory sequences, the locations of the LoxP sites were chosen based on low evolutionary conservation of the DNA sequence between species. The 5′ LoxP site was inserted 671 bp upstream of exon 1. An Frt site flanked neomycin selection cassette immediately followed by the 3′ LoxP site was inserted 1506 bp downstream of exon 1. A 5 kb and a 3 kb 5′ and 3′ homology arms were used, respectively. A thymidine kinase-negative selection cassette was inserted downstream of the 3′ homology arm.

The targeting vector was electroporated in 129SV ES cells and correctly targeted neomycin-resistant colonies were confirmed by long-range PCR (Roche Long template PCR system) using two primer pairs. One pair had a forward primer (5′-GCATCGCATTGTCTGAGTAGGTGTC-3′) located in the neomycin selection cassette and a reverse primer located outside of the 3′ arm of homology (5′-GAAAGAAACTGAAGCAGGGGAGCTGAG-3′). The other pair had a forward primer located outside the 5′ arm of homology (5′-CTCCAGGTCTGTCCTGTGAGGGATG-3′) and a reverse primer that anchored within the 5′ LoxP site[(5′-CATTGGGGGTGGGGTTGACGAATAAC-3′ (ATAAC=LoxP sequence)]. One of several correctly targeted clones with normal karyotypes was used to generate the mouse strain containing the conditional allele. Once germline transmission was achieved, the neomycin selection cassette was excised using *FLPeR* mice (Jackson Laboratories) (Farley et al., 2000). Thus, mice used in the experiments described below have exon 1 of *Trip11* floxed, and no longer have a neomycin cassette. This allele is designated as *Trip11^cko^*, and is genotyped using a primer pair that flanks the 3′ LoxP site (FP, 5′-GGGAAAGACTAGCTAGAGATTGAAC-3′; RP, 5′-TGGCTCTTTACTGGACACATGAAG-3′).

We generated a global *Trip11* knockout allele, here designated as *Trip11*^−^, using the *EIIa-Cre* transgene (Jackson Laboratories) (Lakso et al., 1996) to excise the floxed exon 1. Mice with this *Trip11*^−^ allele are identified using a primer pair with a forward primer located upstream of the 5′ LoxP site (5′-AGTCTCTGGATTTGATCTTCAGCAC-3′) and a reverse primer located downstream of the 3′ LoxP site (5′-TGGCTCTTTACTGGACACATGAAG-3′). Although we targeted *Trip11* in 129SV ES cells, we maintained the *Trip11^cko^* and *Trip11*^−^ alleles by crossing them into C57BL/6 mice.

### Tissue-specific and *ex vivo* inactivation of *Trip11*

We conditionally inactivated *Trip11* in chondrocytes, osteoblasts, hematopoietic stem cells and exocrine pancreatic cells, using *Col2a1*-Cre (kindly provided by Dr Olson, Harvard School of Dental Medicine, Boston, MA, USA), *Bglap*-Cre (Jackson Laboratories, stock # 019509), *Vav1*-Cre (kindly provided by Dr Orkin, Children's Hospital Boston, MA, USA) and *Mist1-CreERT2* (kindly provided by Dr Konieczny, Purdue University, West Lafayette, IN, USA ), respectively (Ovchinnikov et al., 2000; Stadtfeld and Graf, 2005; Tuveson et al., 2006; Zhang et al., 2002). As heterozygous *Trip11* knockout mice are viable, the different Cre alleles were bred to produce Tg:*Col2a1*-Cre;*Trip11*^cko/−^;*ROSA26*^mTmG/+^, Tg:*Bglap*-Cre;*Trip11*^cko/−^;*ROSA26*^mTmG/+^, Tg:*Vav1*-Cre;*Trip11*^cko/−^;*ROSA26*^mTmG/+^ and Tg:*Mist1-*CreERT2;*Trip11*^cko/−^;*ROSA26*^mTmG/+^ mice so that Cre-mediated recombination of only one *Trip11* allele is needed to knockout GMAP-210 function in a cell. Tg:*Col2a1*-Cre;*Trip11*^cko/+^;*ROSA26*^mTmG/+^, Tg:*Bglap*-Cre;*Trip11*^cko/+^;*ROSA26*^mTmG/+^, Tg:*Vav1*-Cre;*Trip11*^cko/+^;*ROSA26*^mTmG/+^ and Tg:*Mist1-*CreERT2*;Trip11*^cko/+^;*ROSA26*^mTmG/+^ littermates served as controls. For inactivation of *Trip11* using the *Mist1-CreERT2* transgene, mice were injected intraperitoneally with tamoxifen (Sigma) (0.1 mg per g of mouse weight; tamoxifen stock: 10 mg/ml in corn oil) three times with the injections separated by 1 day. The *ROSA26*^mTmG^ allele (Jackson laboratories, stock # 007576) was used to visualize and/or sort Cre-recombined cells based on their having enhanced green fluorescence protein expression (Muzumdar et al., 2007). In order to inactivate *Trip11* in primary chondrocyte pellet cultures *ex vivo*, we used Tg:*Cag-*Cre/Esr1 mice (Jackson laboratories, stock # 004682) (Hayashi and McMahon, 2002) to produce offspring with the genotype *Tg:Cag-*Cre/Esr1;*Trip11^cko/−^;ROSA26*^mTmG/+^ from which we isolated chondrocytes.

### Skeletal preparation

Alcian Blue and Alizarin Red staining of intact skeletons were performed as previously described (Smits et al., 2004, 2001). In short, embryos or newborn pups were humanely sacrificed and placed in hot tap water for 10 min. The skin was carefully removed using forceps and the animals were eviscerated. Fixation was carried out in 95% ethanol for 3 days, after which the bodies were stained in Alcian Blue staining solution [0.015% Alcian Blue 8GX (Sigma), 20% acetic acid, 76% ethanol] for 24 h. The bodies were subsequently washed three times with 95% ethanol (24 h for each wash). Bodies were cleared in 1% KOH for 24 h and stained for 12 h in Alizarin Red staining solution [0.005% Alizarin Red (Sigma), 2% KOH]. Bodies were then cleared in 2% KOH for 24 h. The clearing process was completed using the following ratios of 2% KOH to glycerol (80:20, 60:40, 40:60 and 20:80). Each clearing step was performed for 24 h. Pictures of the stained skeletons were taken using NIS-element AR 4.20.01 software (Nikon), using a Nikon SMZ18 stereomicroscope (10× objective, 0.75× lens) equipped with a Nikon digital sight DS-Ri1 camera.

### Histology

Forelimbs of embryos and pups were fixed with 4% paraformaldehyde in PBS for 24 h and subsequently demineralized for 7 days in 10% EDTA. Samples were dehydrated using a graded series of ethanol (30%, 50%, 70%, 95% and twice in 100%; 12 h for each step). Samples were then cleared with xylene (twice for 45 min) and saturated in paraffin (twice for 1 h). After embedding, 7 µm sections were cut.

For Alcian Blue staining, sections were deparafinized with xylene (twice for 3 min), rehydrated using a graded series of ethanol (twice in 100% for 1 min, then once in 95%, 70%, 50% and 30% ethanol for 30 s). Sections were placed in 3% acetic acid for 3 min and stained in Alcian Blue staining solution [1% Alcian Blue 8GX (Sigma) in 3% acetic acid] for 30 min. Next, sections were washed for 10 min under running tap water and counterstained with Nuclear Fast Red [0.1% Nuclear Fast Red (Sigma) in 5% aluminum sulfate] for 5 min. Subsequently the sections were washed under running tap water for 1 min and dehydrated with a graded series of ethanol (once at 30%, 50%, 70% and 95% ethanol for 30 s followed by twice in 100% ethanol for 1 min). Sections were then cleared with xylene (twice for 1 min), dried and mounted using Permount (Sigma).

Pictures of the stained sections were taken using NIS-element AR 4.20.01 software (Nikon), using a Nikon Eclipse 80i microscope (10× objective combined with a 10× or 20× lens) equipped with a Nikon digital sight DS-Ri1 camera. Images of control and mutant samples were adjusted identically for brightness, contrast and sharpness (Adobe Photoshop CS6).

### μCT measurements

μCT measurements of tibia mid-shaft cortical bone and proximal tibial trabecular bone were performed as previously described (Cui et al., 2011; Sawakami et al., 2006). Briefly, the right tibiae from 6- to 8 week-old male mice were dissected from each carcass and preserved in 70% ethanol for μCT scanning on a Scanco Medical μCT 35 system. Specimens from mice with the *Bglap*-Cre transgene were scanned with an isotropic voxel size of 10 µm at 50 kV tube voltage, 120 mA and a 151 ms integration time and the regions of interest depicted in Fig. 7B were analyzed. Specimens from mice with the *Vav1-*Cre transgene were scanned with an isotropic voxel size of 7 μm using an X-ray tube potential of 55 kVp, an X-ray intensity of 0.145 mA and an integration time of 600 ms. A region beginning 1.4 mm distal to the proximal growth plate and extending 1.4 mm distally was selected for trabecular bone analysis. A second region 3.0 mm proximal to the tibia-fibula junction and 0.7 mm in length was selected for cortical analysis. A semi-automated contouring approach was used to distinguish cortical and trabecular bone. 3D renderings were generated and microstructural properties of bone were calculated using software supplied by the manufacturer.

### Electron microscopy

Samples were fixed at 4°C for 48 h in 2% formaldehyde, 2.5% glutaraldehyde and 0.1 M sodium cacodylate buffer (pH 7.4), decalcified for 1 week using 10% EDTA, and re-fixed for 24 h. After fixation and decalcification, specimens were treated with 1% osmium tetroxide, 1% uranyl acetate in maleate buffer, dehydrated in a graded ethanol series, treated with propylene oxide and embedded in Taab epon mixture at 60°C. Blocks were sectioned at 95 nm on a Leica Ultracut microtome and viewed and imaged with a Philips Tecnai Spirit electron microscope (2900× and 93,000×). Images displayed were adjusted for brightness and contrast to offset bleaching (Adobe Photoshop CS6).

### Cultivation of primary mouse embryonic fibroblasts

Mouse embryonic fibroblasts were prepared using E13.5 *Trip11^+/+^, Trip11^cko/+^* and *Trip11^−/−^* embryos. Yolk sacs were used to confirm the genotypes. The head and internal organs were removed, then each body was placed in a well of a 24-well plate immersed in 1 ml 0.25% trypsin-EDTA (Life Technologies). Disaggregation was achieved by pulling each embryo through an 18 G needle five times and then digesting for 30 min at 37°C in a 5% CO_2_ incubator. Cells were vigorously pipetted to obtain a single cell solution, which was transferred to 20 ml DMEM/10% FBS with DNAse I (Roche) added to a final concentration of 100 µg/ml and incubated at 37°C for 15 min. Cells were then pelleted, washed in PBS and plated in DMEM/10% FBS on gelatinized 10 cm dishes (one embryo/dish).

### Cultivation and cell sorting of primary osteoblasts

Five-day-old Tg:*Bglap*-Cre;*Trip11*^cko/+^*;ROSA26*^mTmG*/+*^ and Tg:*Bglap*-Cre;*Trip*^cko/−^*;ROSA26*^mTmG/+^ littermates were decapitated and their heads placed in sterile PBS. Parietal calvarial bone was recovered and cleaned. After rinsing three times in PBS, the bone was serially placed in digestion medium [0.625% collagenase II (Life Technologies)/0.01% Trypsin (Fisher) in PBS] three times, for 15 min each at 37°C. Calvaria were moved to 1 ml growth medium [DMEM containing 10% fetal bovine serum (GemCell), 100 U/ml penicillin (Life Technologies), 100 µg/ml streptomycin (Life Technologies)]. Pre-osteoblasts were allowed to grow out of the calvaria and were cultured and expanded until they became confluent in 75 cm^2^ culture flasks. Single cell suspensions were then made using digestion medium and GFP-expressing cells were recovered by flow sorting (BL2-Aria IIU cell sorter).

### Extraction of bone marrow hematopoietic precursors

Eight-week-old Tg:*Vav1*-Cre;*Trip11*^cko/+^;*ROSA26*^mTmG/+^ and Tg:*Vav1*-Cre;*Trip11*^cko/−^;*ROSA26*^mTmG/+^ littermates were euthanized and their tibias and femurs recovered, washed three times in PBS containing 1000 U/ml penicillin (Life Technologies) and 1 mg/ml streptomycin (Life Technologies), and then placed in regular PBS. The epiphyses of the bones were cut off and the metaphyses were flushed with 10 ml of PBS using a 25 G needle. The flushed cells were filtered through a 48 µm cell strainer and then collected by centrifugation (400 ***g*** for 5 min). Pelleted cells were re-suspended in 10 ml growth medium [α-MEM containing 10% fetal bovine serum (GemCell), 100 U/ml penicillin and 100 µg/ml streptomycin] and plated in 10 cm^2^ culture dishes. Fibroblasts were allowed to attach for 12 h to the culture dish at 37°C in a 5% CO_2_ incubator after which non-adherent cells were collected, washed with PBS and used for western blotting.

### Primary chondrocyte pellet cultures

Seven-day-old *Trip11^cko/cko^*;*ROSA26*^mTmG/+^ and Tg:*Cag*-Cre/Esr1;*Trip11*^cko/−^;*ROSA26*^mTmG/+^ mice were euthanized, and their ribcages recovered and washed three times with 2 ml PBS containing 1000 U/ml penicillin (Life Technologies) and 1 mg/ml streptomycin (Life Technologies). Ribcages were cleaned of adherent tissue by incubating them in 2 ml digestion medium [DMEM/F12 (Life Technologies) containing 3 mg/ml collagenase A (Roche), 100 U/ml penicillin and 100 µg/ml streptomycin] at 37°C for 1 h in a 5% CO_2_ incubator. Ribcages were then washed once with PBS, transferred to new digestion medium and incubated at 37°C in a 5% CO_2_ incubator. At 4 h and 6 h, pipetting was performed to release cells from the digesting tissue. The single cell suspension was filtered through a 48 µm cell strainer into a 50 ml conical tube containing 10 ml complete medium [DMEM/F12 containing 10% fetal bovine serum (GemCell), 100 U/ml penicillin, 100 µg/ml streptomycin and 50 µg/ml ascorbic acid (Sigma)]. Cells were pelleted (400 ***g*** for 5 min), washed in PBS, repelleted and resuspended in complete medium containing 4-OH-tamoxifen (Sigma) at a final concentration of 1 µM. Cells were again pelleted, the cap of the 14 ml tube was loosened and the cell pellets were cultured at 37°C in a 5% CO_2_ incubator for 14 days under continuous 4-OH-tamoxifen exposure. Medium was replaced every other day. After 14 days, pellets were dissociated by incubating them for 1 h in digestion medium. Cells were washed once with PBS and used for mass spectrometry and western blot.

### Western blotting

Cells were lysed on ice in 1 ml mammalian lysis buffer (Promega) containing 1× protease inhibitor cocktail (Promega) for 10 min. Protein concentration was measured using the Bio-Rad protein DC assay. Ten µg of each sample was separated on a 3-8% Tris-acetate SDS PAGE gel (Life Technologies) and transferred to Invitrolon 0.45 µm pore PVDF membranes (Life Technologies). Membranes were blocked for 30 min in western breeze blocking buffer (Life Technologies). Primary antibodies were diluted in western breeze antibody dilution buffer and incubated with the membranes for 1 h at room temperature. Membranes were then washed three times for 5 min each with western breeze wash buffer. Peroxidase-coupled secondary antibodies were diluted in western breeze antibody dilution buffer and incubated with the membranes for 1 h at room temperature followed by three washes. Membranes were rinsed twice with water and incubated for 5 min with Tropix CDP star substrate (Applied Biosystems). Immunoreactive bands were visualized using Hyblot CL autoradiography film (Denville).

Primary antibodies used were: anti-TRIP11/GMAP-210 10G5 (LifeSpan Biosciences, LS-C20059) at a 1/500 dilution; anti-HSPG2 A76 (Abcam, ab26265) at a 1/500 dilution; anti-COL9A2 (Santa Cruz, sc-398130) at a 1/500 dilution; anti-ACTIN AC-15 (Sigma, A1978) at a 1/15,000 dilution; and anti-tubulin (Sigma, T8203) at a 1/1000 dilution. Secondary antibody used was peroxidase-coupled goat anti-mouse IgG (Thermo Scientific, 31320) at a 1/10,000 dilution.

### SDS-PAGE of plasma samples

Blood was extracted using a 1 ml syringe with an 18 G needle from the heart of euthanized mice immediately after death. Blood samples were collected in 1 ml MiniCollect Lithium/Heparin tubes (Greiner-bio), spun down at 400 ***g*** for 15 min to separate serum from plasma. Plasma aliquots were added to 1× SDS-PAGE loading buffer/denaturation solution (Thermo Scientific), denatured at 70°C for 10 min and separated on a 3-8% Tris-acetate SDS-PAGE gel (Thermo Scientific). After electrophoresis, gels were washed three times for 5 min with water, stained with Coomassie Blue (SimplyBlue Safe stain, Thermo Scientific) and destained with deionized water for 24 h.

### Sample preparation, quantitative mass spectrometry and data analysis

Chondrocyte cell pellets were resuspended in lysis buffer [8 M urea, 150 mM NaCl, 200 mM EPPS (pH 8.5)] with complete protease inhibitors (Roche), and lysed by syringe pumping (*n*_pumps_=10) cells through a 25(5/8) gauge needle. Protein lysates were reduced (5 mM TCEP) and alkylated (15 mM iodoacetamide) before chloroform/methanol precipitation of the protein lysate. Proteins were resuspended in 200 mM EPPS (pH 8.5) and digested with LysC overnight (Wako, room temperature), followed by a 6 h trypsin digestion (Promega, 37°C). For each biological replicate, 100 µg of peptide was labeled with tandem mass tag (TMT, Thermo Scientific) reagents at a 2:1 (w/w) TMT:peptide ratio. After a 1.5 h at room temperature, the reaction was quenched (0.5% hydroxylamine) and the peptide samples were mixed. Mixed samples were fractionated by high-pH reverse phase chromatography for a final total of 12 pooled fractions spanning the gradient (Yang et al., 2012). Fractionated peptides were desalted by in-house made C_18_ stage-tips and loaded on an in-house pulled C_18_ analytical column (35 cm total length with 100 μm, 2.6 Å beads) before being injected into an Orbitrap Fusion Lumos mass spectrometer running an LC-MS/MS/MS top-10 data-dependent method using synchronous-precursor-selection-MS^3^ for TMT quantification (McAlister et al., 2014). Peptide-spectral matches were determined using SEQUEST, which identified 102693 total peptides across 12 pooled fractions (Eng et al., 1994). Peptide spectral matches were filtered to a peptide and protein false discovery rate (FDR) both less than 1%. Significance of protein quantitative results was determined based on a Student's *t*-test, and multiple test correction of *P*-values was carried out according to Benjamini-Hochberg FDR less than 0.05 (Benjamini and Hochberg, 1995). Data analysis and quantitative proteomics plotting was carried out using the R programming language (www.R-project.org/).

## Supplementary Material

Supplementary information

Supplementary information
