# Supplementary material for: The skeletal phenotype of achondrogenesis type 1A is caused exclusively by cartilage defects
Source: Development. 2018 Jan 1;145(1):dev156588. doi: 10.1242/dev.156588 (PMC5825869; doi:10.1242/dev.156588)
Supplement: Supplementary information [file develop-145-156588-s1.pdf]

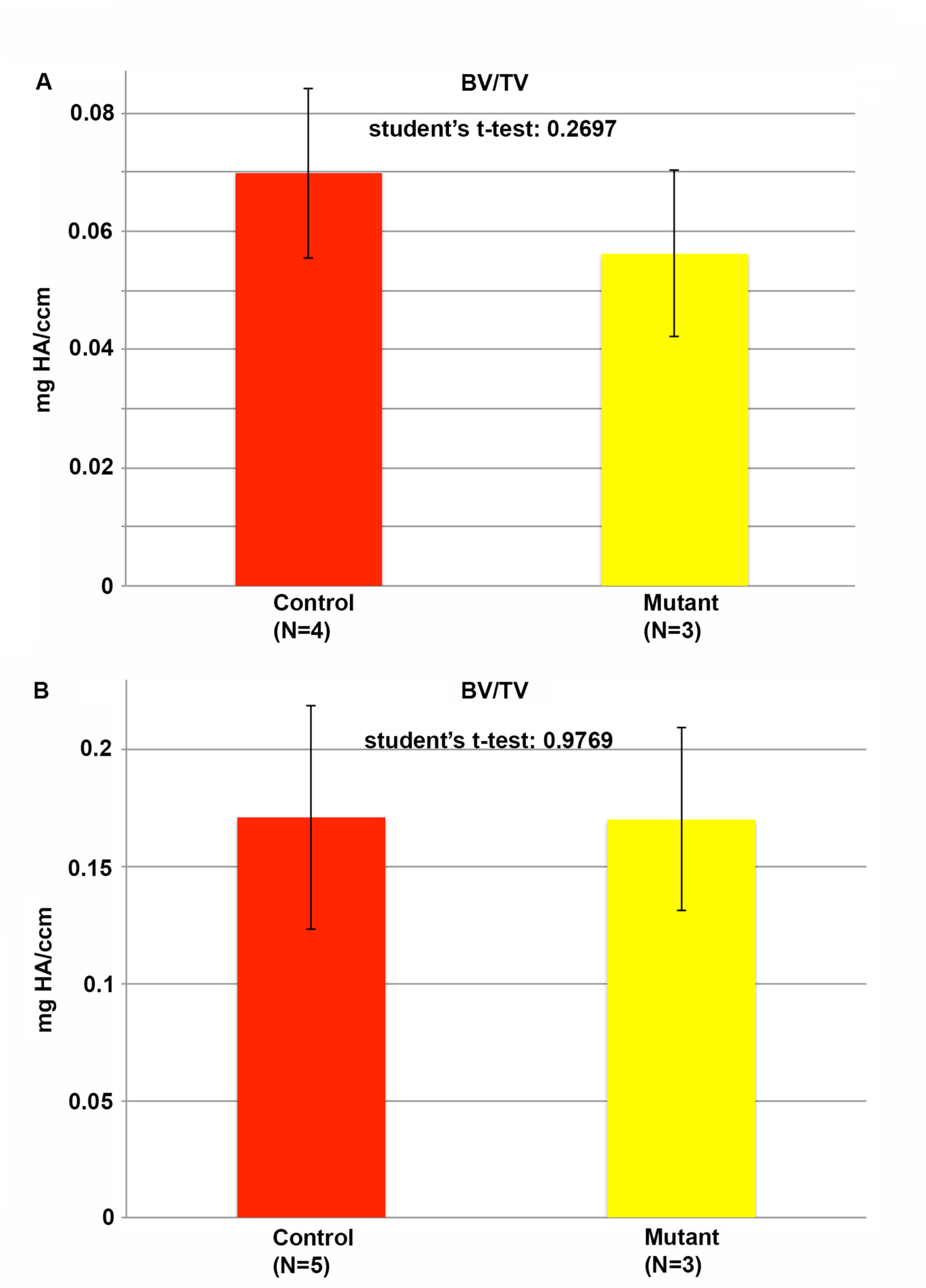

Development • Supplementary information

Supplemental Figure 1: Absence of GMAP-210 in osteoblasts or osteoclasts does not affect bone formation. **1A)** Trabecular BV/TV µCT measurements of the tibia of male control (4 Tg:*Bglap*-Cre;*Trip11*$^{cko/+}$*;ROSA26*$^{mTmG/+}$) and osteoblast knockout (3 Tg:*Bglap*-Cre;*Trip11*$^{cko/-}$*;ROSA26*$^{mTmG/+}$) mice. **1B)** Trabecular BV/TV µCT measurements of the tibia of male control (three *Trip11*$^{cko/-}$*;ROSA26*$^{mTmG/+}$ and two Tg:*Vav1*-Cre;*Trip11*$^{cko/+}$*;ROSA26*$^{mTmG/+}$) and hematopoietic knockout (Tg:*Vav1*-Cre;*Trip11*$^{cko/-}$*;ROSA26*$^{mTmG/+}$) mice. Note the absence of a significant difference in BV/TV for both knockout models.

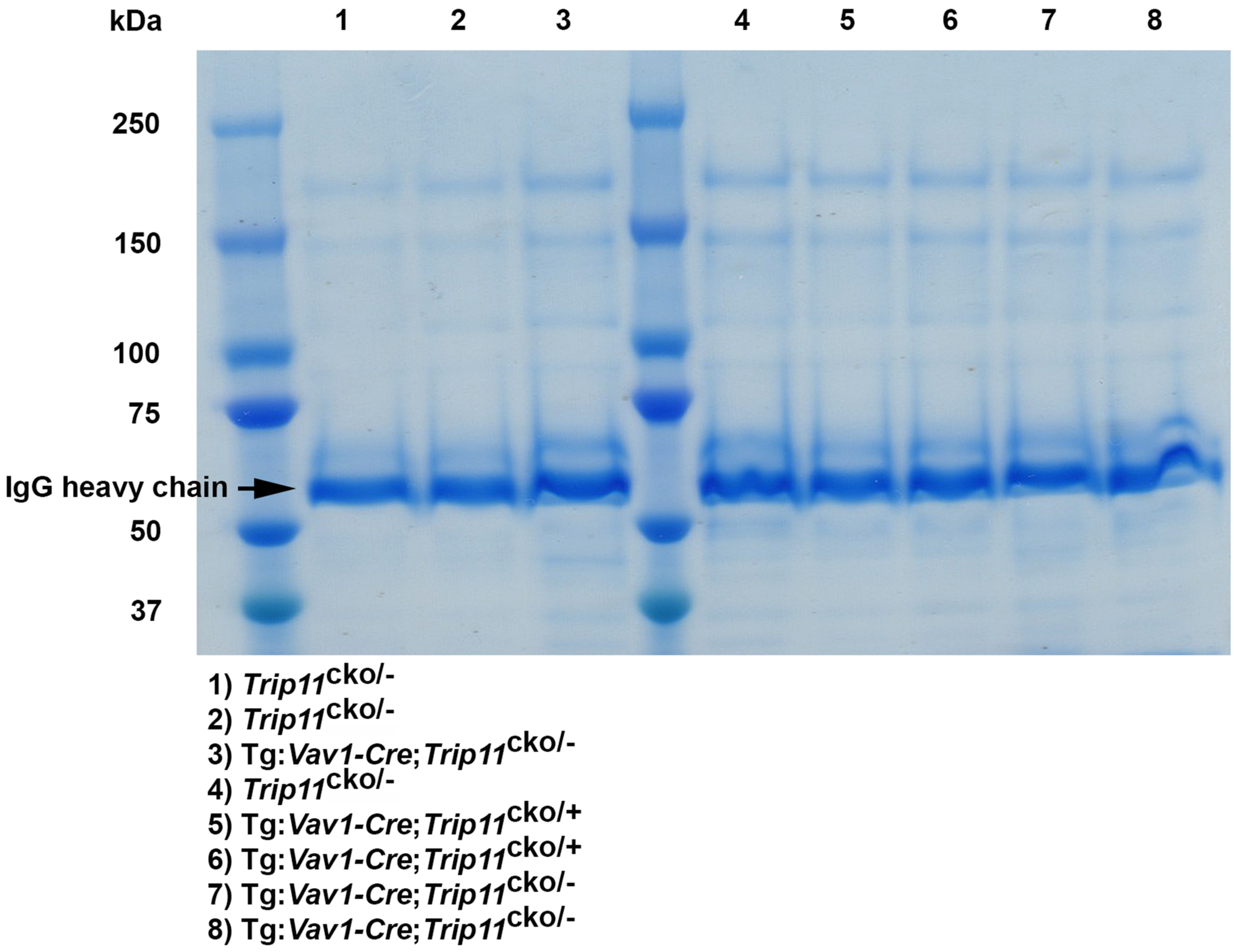

Supplemental Figure 2: Absence of GMAP-210 in lymphocytes does not interfere with IgG secretion. 0.1 ul of serum, extracted from 8-week old mice of the indicated genotypes, was separated on a 3-8% Tris-Acetate SDS-PAGE gel and stained with Coomassie blue. Note that there is no difference in staining intensity of the IgG heavy chain band between control (1, 2, 4, 5 and 6) and mutant (3, 7 and 8) mice. (litter 1: samples 1, 2 and 3; litter 2: samples 4, 5, 6, 7 and 8).

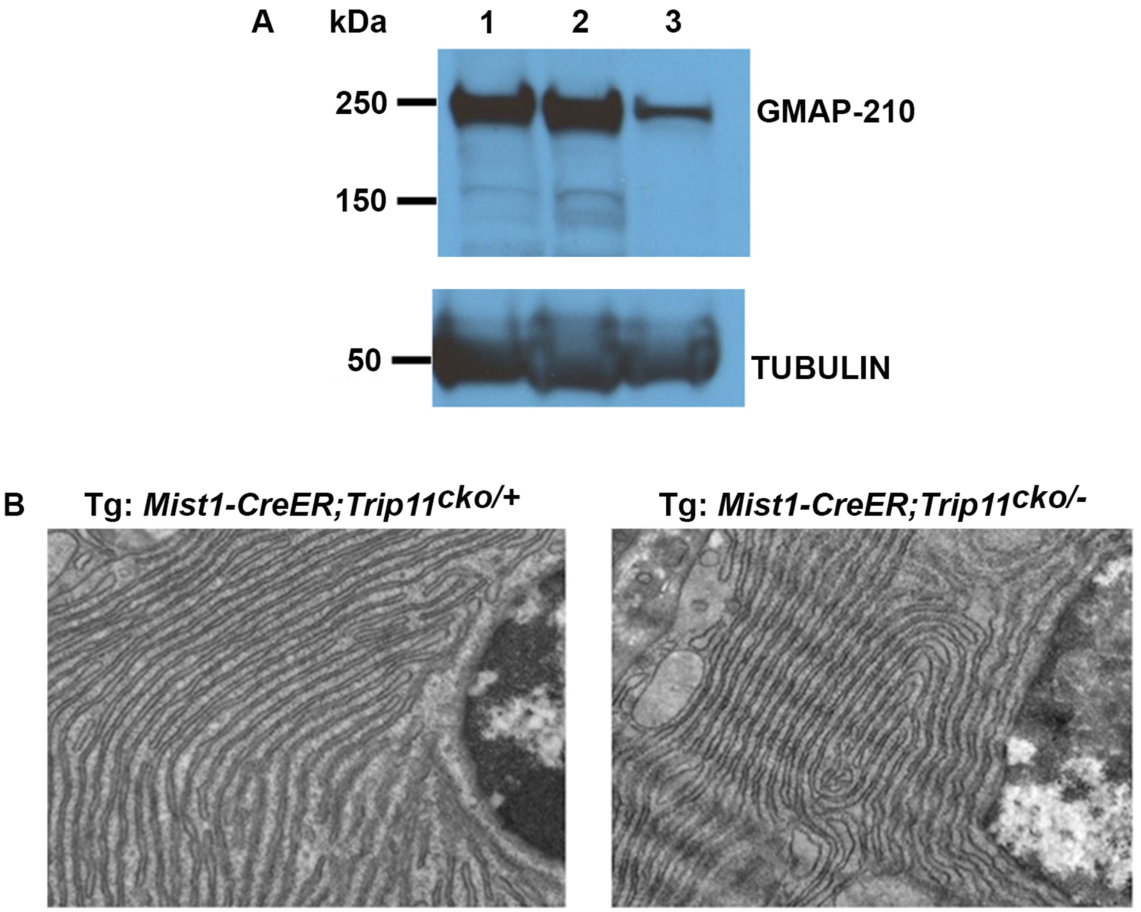

Supplemental Figure 3: Specific inactivation of *Trip11* in the exocrine acinar cells of the pancreas does not result in swelling of ER cisternae. A) Western blot analysis of lysates generated from the pancreas of 8-week old control (1 and 2: *Tg:Mist1-CreER;Trip11*$^{cko/+}$*;ROSA26*$^{mTmG/+}$) and acinar cell knockout (3: *Tg:Mist1-CreER;Trip11*$^{cko/-}$*;ROSA26*$^{mTmG/+}$) mice, 1 week after tamoxifen treatment. Note the reduction in GMAP-210 protein levels in the acinar cell knockout. **B)** Transmission electron microscopy picture of ER cisternae of acinar cells from 12 week-old control (*Tg:Mist1-CreER;Trip11*$^{cko/-}$*;ROSA26*$^{mTmG/+}$) and acinar cell knockout (*Tg:Mist1-CreER;Trip11*$^{cko/-}$*;ROSA26*$^{mTmG/+}$) mice, 1 month after their tamoxifen treatment. Magnification 6800x. Note the absence of swelling of ER cisternae in control and mutant mice. N=3, one representative result is shown.

Development • Supplementary information

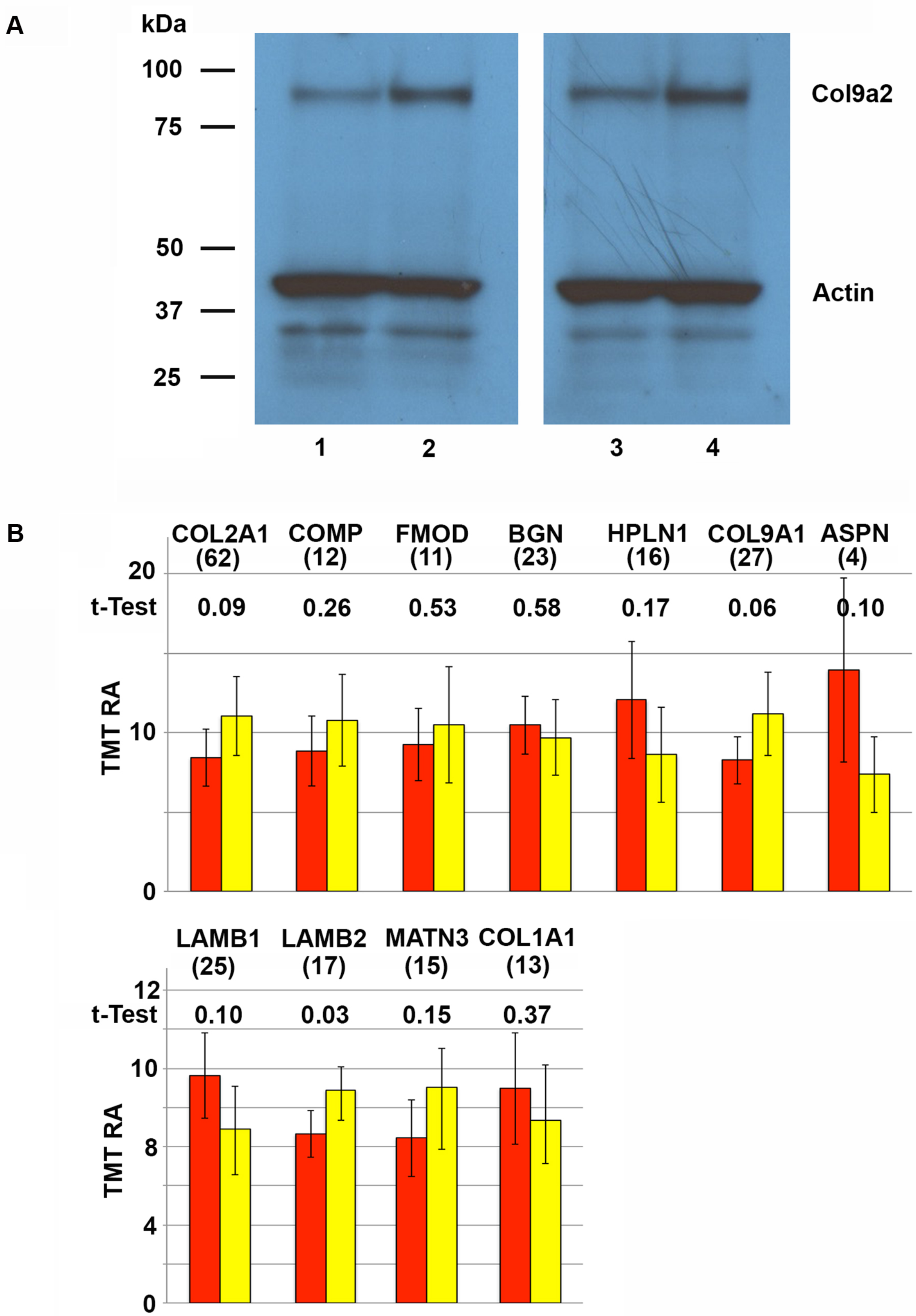

Development • Supplementary information

Supplemental Figure 4: *Ex vivo* inactivation of *Trip11* in primary chondrocyte pellet cultures. A) Accumulation of COL9A2 in GMAP-210 depleted chondrocytes. Western blot of cell lysates from 4-OH tamoxifen treated primary chondrocyte pellet cultures using antibodies against COL9A1 and actin (loading control). Lysates were generated from two separate pellet culture experiments. Lanes 1 and 3: control pellet cultures (*Trip11*$^{cko/cko}$*;ROSA26*$^{mTmG/+}$); Lanes 2 and 4: 4-OH-tamoxifen induced *Trip11* knockout pellet cultures (Tg:*CagCre/Esr1;Trip11*$^{cko/-}$*;ROSA26*$^{mTmG/+}$). Note the increase in immuno-detectable COL9A2 in the *Trip11* inactivated samples. **B) Absence of GMAP-210 does not result in the intracellular accumulation of most extracellular matrix proteins.** Abundance of non-significantly affected extracellular matrix proteins in the lysates of 4-OH tamoxifen treated control (*Trip11*$^{cko/cko}$*;ROSA26*$^{mTmG/+}$) (red) and mutant (Tg:*CagCre/Esr1;Trip11*$^{cko/-}$*;ROSA26*$^{mTmG/+}$) (yellow) chondrocyte pellet cultures as determined by tandem tag mass spectroscopy. T-Test: student's t-test adjusted p-value. B/H: Benjamini-Hochberg adjusted p-value. TMT-RA : Tandem mass tag relative abundance. (COL2A1: Type 2 collagen α1 chain; COMP: Cartilage oligomeric matrix protein; FMOD: Fibromodulin; BGN: Biglycan; HPLN1: Cartilage link protein; COL9A1: Type 9 collagen α1 chain; ASPN: Asporin; LAMB1-2: Laminin beta 1-2; MATN3; Matrillin 3; COL1A1: Type 1 collagen α1 chain)

Development • Supplementary information

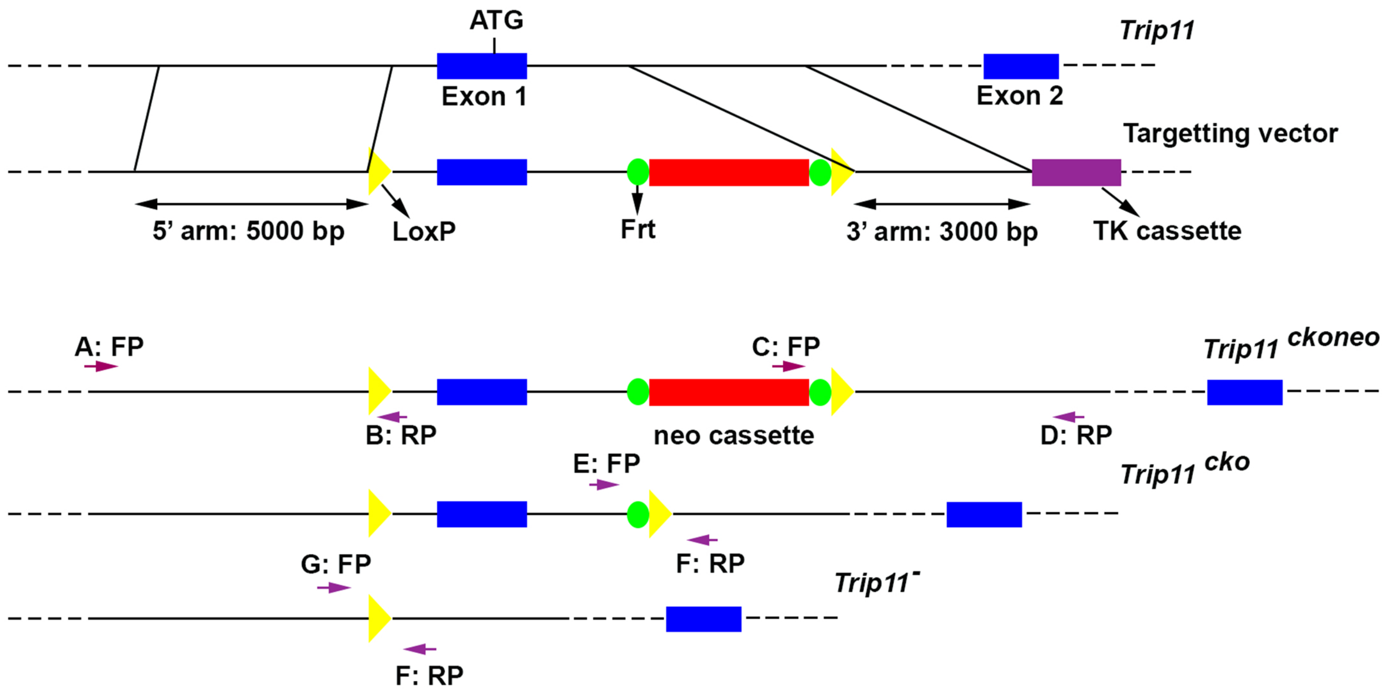

Supplemental Figure 5: Schematic representation of the method used to generate the *Trip11* conditional (*Trip11*^{cko}) and knockout (*Trip11*$^{-}$) alleles. The *Trip11*^{cko} targeting vector contained the following features from 5' to 3': a 5000 bp 5' homology arm; a 5' LoxP site located 672 bp upstream from the start of the ATG containing first exon of *Trip11*; a 2661 DNA fragment (-671 to +1989, 0 = start of transcription) containing the first exon; a FRT flanked neomycin positive selection cassette; a 3' LoxP site located 1989 bp downstream of the start of transcription; a 3000 bp 3' homology arm and a Thymidine Kinase (TK) negative selection cassette. Homologous recombination in ES cells generated the *Trip11*$^{cko\text{-}neo}$ allele. After generation of chimeric mice and germline transmission of the *Trip11*$^{cko\text{-}neo}$ allele, the conditional (*Trip11*^{cko}) allele was generated by the removing the neo cassette using *FLPeR* mice. Finally a knockout allele (*Trip11*$^{-}$) was generated using *EIIa-Cre* mice. Arrows indicate locations of PCR primers used for genotyping the different alleles.

Development • Supplementary information

Supplemental Table 1: All LC-MS/MS/MS identified and quantified proteins and signal-to-noise values for the quantified TMT channels.

Click here to Download Table S1

Supplemental Table 2: LC-MS/MS/MS identified and quantified proteins with significantly changed protein abundances between the *Trip11* knockout and control cells.

Click here to Download Table S2
